# CCC meets ICU: Redefining the role of critical care of cancer patients

**DOI:** 10.1186/1471-2407-10-612

**Published:** 2010-11-08

**Authors:** Michael von Bergwelt-Baildon, Michael J Hallek, Alexander A Shimabukuro-Vornhagen, Matthias Kochanek

**Affiliations:** 1 Department I of Internal Medicine, University of Köln, Kerpenerstrasse 62, 50937 Cologne, Germany; 2 Center for Integrated Oncology Köln-Bonn, University of Köln, Kerpenerstrasse 62, 50937 Cologne, Germany

## Abstract

**Background:**

Currently the majority of cancer patients are considered ineligible for intensive care treatment and oncologists are struggling to get their patients admitted to intensive care units. Critical care and oncology are frequently two separate worlds that communicate rarely and thus do not share novel developments in their fields. However, cancer medicine is rapidly improving and cancer is eventually becoming a chronic disease. Oncology is therefore characterized by a growing number of older and medically unfit patients that receive numerous novel drug classes with unexpected side effects.

**Discussion:**

All of these changes will generate more medically challenging patients in acute distress that need to be considered for intensive care. An intense exchange between intensivists, oncologists, psychologists and palliative care specialists is warranted to communicate the developments in each field in order to improve triage and patient treatment. Here, we argue that "critical care of cancer patients" needs to be recognized as a medical subspecialty and that there is an urgent need to develop it systematically.

**Conclusion:**

As prognosis of cancer improves, novel therapeutic concepts are being introduced and more and more older cancer patients receive full treatment the number of acutely ill patients is growing significantly. This development a major challenge to current concepts of intensive care and it needs to be redefined who of these patients should be treated, for how long and how intensively.

## Background

The discovery of the human genome and the development of high-power bioinfomatics tools have led to an exponential growth of biomedical knowledge. One of the areas that has benefited the most over the last few years has been cancer research which is now translating into a dramatic development of clinical oncology. As a result targeted therapy and personalized medicine will eventually transform cancer into a chronic or even curable disease.

While improved overall survival and reduced acute toxicities are already visible in certain, defined clinical scenarios [1-3], secondary consequences such as long-term toxicities, quality of life, long-term cancer survivorship, the role of palliative care etc. need to be addressed in novel research programs. The growing complexity of cancer medicine has led to the development of comprehensive cancer centers, institutions designed to bring multi-disciplinary care to the patient ensuring all important angles of diagnostics and therapy are covered.

Here, we propose that one of the most striking consequences is the urgent need for new critical care concepts for cancer patients. This hypothesis is supported by the following arguments:

Some cancers are likely to become a *chronic disease*. However, in accordance with current recommendations advanced stage cancer patients are frequently denied admission by intensive care units that are normally run by non-oncologists [4-6]. When a given patient's life expectancy increases from three months to three years these concepts are radically challenged: How many ICU or respirator days are reasonable? How aggressive should such a treatment be: Is non-invasive ventilation the maximum support or can select cases be identified where extracorporeal respiratory devices such as ECLA (extra corporal lung assist device) or ECMO (extra corporal membrane oxygenization) will be a reasonable choice? Is a five-agent-vasopressor-therapy in principle not advisable for cancer patients or should septic shock in a slowly progressive colon cancer patient on bevacizumab/capecitabine with an ECOG-1 (Eastern Cooperative Oncology Group) performance since diagnosis five years ago receive extensive treatment?

When critical care comes into play does *palliative care *need to step back or even come closer? In terminally ill lung cancer patients for example the frequency of end-of-life-admissions to hospices as well as ICUs have been reported to increase substantially suggesting that both specialities are needed for this patient cohort [7]. Early involvement of palliative care teams in the treatment of pediatric cancer patients has demonstrated to reduce unnecessary full code orders and ease suffering [8]. This emphasizes the rarely appreciated interphase between two distinct medical specialities that must enter into a continuous dialogue to develop a culture of common patient-family discussions, consultations and optimization of treatment practice.

In cancer patients neutropenia, autologous bone marrow transplantation and the characteristics of the underlying malignancy now have limited influence on the acute outcome while the number of organ failures and severity of infection are correlate with survival [9-12]. ICU survival of cancer patients has been shown to improve over time [13-16]. Even survival after cardiopulmonary resuscitation of cancer patients compares favorably to unselected patients [17]. However, triage of critically ill cancer patients using classic *scoring systems *appears to be difficult as they are not developed for this cohort and often lead to wrong predictions. Therefore, they are the subject of an ongoing debate and further improvement appears necessary [18-22]. Even cancer-specific scores such as the Cancer Mortality Model are not widely used as they appear not to improve mortality prediction [20,22].

This is of particular importance as the treating *physician's perception *of a critically ill patient's prognosis has been shown to influence survival more strongly than the baseline disease severity, development of organ dysfunction or use of vasopressors [23]. The prognosis of critically ill cancer patients is generally perceived to be extremely poor by most physicians. Consequently, critically ill cancer patients might often be refused intensive care. However, a recent study showed a high mortality in ill cancer patients who were considered to be too well for ICU admission while patients who were considered to be too sick could benefit from ICU admission [24]. A reappraisal of ICU admission criteria for cancer patients is therefore required. Triage decisions solely based on the type of cancer are thus not justified but instead should be made by an interdisciplinary team consisting of intensivists, medical and surgical oncologists with experience in treating critically ill cancer patients.

Life expectancy is rising globally and in 2025 1.2 billion people will be older than 60 years of age [25]. Therefore, more and more older people will be treated long-term for cancer bringing clinical problems of *internal and geriatric medicine *into the world of cancer treatment [26]. So far patients with significant co-morbidities or higher age are rarely treated intensively and complex operations, high-dose chemotherapies and stem cell transplantation are classically strategies for the young and fit. Reduced-intensity conditioning for allogeneic BMT is only one example of a development that will bring medical conditions such as renal, cardiac or vascular insufficiency to the oncology wards and thus more patients with such conditions and cancer to the ICU. How do we treat a sepsis in a neutropenic patient that harbours several foreign bodies such as pace makers, PTFD shunts and total endoprotheses? How do we dose novel targeted drugs in patients with renal or hepatic failure undergoing SLEDD (sustained slow-efficiency dialysis) or MARS (Molecular Adsorbents Recirculation System) treatment? How to dose anticoagulation in patients with atrial fibrillation and thrombopenia on prothrombotic biologicals such as lenalidomide?

These issues can only be addressed when medical oncology and geriatric as well as intensive care medicine start working closely together to develop common, interdisciplinary algorithms.

An increasing number of *novel drug classes *is entering the market and novel, unexpected serious adverse events (SAE) are encountered [27-29]. A closer collaboration between oncology wards and ICUs e.g. by using shared beds might be a good environment to monitor and treat patients receiving novel agents or dose levels. No intensivist alone can anticipate rare side effects without an in-depth knowledge of oncological drug actions. On the contrary oncologists will hardly be able to manage severe adverse effects they have seen twice in their lifetime. Managing these problems together will not only be safest for the patient but will also accelerate the understanding of drug/patient interactions.

To date future intensive care patients frequently enter the hospital via the emergency room in critical conditions necessitating sedation and respiratory support. A personal relationship to the ICU staff is therefore only developed in select cases. Cancer patients are treated long-term and *personal relationships with the treating nurses and physicians *are common. If such patients enter the ICU the physician-patient relationship becomes extraordinarily difficult as the ICU days mean much more suffering and pain than regular treatment. It is hard to imagine that this stress can be tolerated by the caregivers without long-term psychological damage or at least specialized psychological guidance. ICUs specialized in the challenges of cancer medicine would have the opportunity to develop psychological support strategies so far not existent in critical care.

The dynamic of this development is underlined by the fact that substantial progress has already been made both in intensive care and hematology/oncology which already results in an improved survival of critically ill cancer patients [30-32].

## Discussion

To best address the above challenges we propose a four step strategy:

1. Permissive ICU admission policies for cancer patients and *early and aggressive treatment *has been reported to be beneficial for cancer and non-cancer patients alike [30,33,34]. Development of organ failure has been shown to have a good predictive value concerning the mortality [24,30,35]. Therefore, rapid and comprehensive initiation of treatment should be followed by a defined treatment phase which will allow to observe development of disease severity, identify further oncological treatment options and determine the prognosis of the acute disease. After a defined period e.g. 4-6 days an interdisciplinary meeting consisting of oncologists, intensivists, nurses, psychologists, palliative care and ethics specialists should be held to reevaluate the situation and possibly redefine the treatment goals. In most situations comprehensive interdisciplinary decision making is not possible in the phase of ICU admission due to occurrence during after hours or the need to act immediately in emergencies. A *reevaluation after a limited treatment phase *appears to allow for more solid judgment.

2. Oncologists spend more time talking to the patients and their families than colleagues from many other specialties. However, the aspect of intensive care and resuscitation are rarely addressed in these discussions. Therefore, end-of-life decisions and do-not-resuscitate orders are often issued by the treating intensivist alone [36,37]. This is rather suboptimal as these decisions often have to be made when the patient (and the family) is barely able to participate in such a discussion and the treating physician is under considerable stress (night time work, urgency of situation, suffering patient etc.). We suggest that the treating oncologist should *address ICU admission and code orders early *to learn the patient's and family's opinion and to give them time to consider. This information should help define preliminary treatment goals upon admission and refine them after an early treatment phase of 4-6 days.

3. How can oncological knowledge best be brought to the ICU and how can critical care be optimized for cancer patients? The already growing number of cancer patients undergoing critical care currently necessitates a 'distribution' of these patients to IC wards of various specialties. This development is viewed with concern, not only by the patients and their families, but also by the responsible physicians, and is certainly not a desirable development.

Oncologists and intensivists are usually distinct types of physicians that clearly know why they do not want to perform their colleagues' work. While oncologist have significant time to research and identify the optimal strategies for their patients, intensivists sometimes need to make quick decisions based on little or -on the contrary-, extremely complex, but inconclusive information. Oncologists spend a lot of their time talking with patients and families while intensivists focus on delicate, invasive procedures. Therefore, they share little common ground and patients either benefit from the one set of skills or the other, rarely both at the same time.

The most obvious solution would be to have common rounds and prime different mind sets by common rotations during training. Intensivists should be encouraged to acquire practical and theoretical knowledge of haematology and oncology as part of their specialty training and vice versa.

An alternative approach is being pursued at our institution where a medical ICU (MICU) is integrated into the oncology service. The department of medical oncology is part of a regional comprehensive cancer center and the MICU part of a portal for emergency medicine and intensive care. Thus there are closest interactions with neighboring specialties e.g. radiotherapy, gastroenterology, palliative care, surgery, psychology on the one hand and anesthesia, emergency medicine, general as well as specialized surgical, neurological and cardiac intensive care on the other. The ICU is directed by physicians that are trained medical oncologists and intensive care specialists. An increasing number of interns that were once dedicated to become oncologists have now discovered their interest and talent for the other specialty and are pursuing such a double qualification. This is a development we have anecdotally seen in the field of bone marrow transplantation where haematologist had become familiar with the treatment of their critically ill patients due to isolation policies. However, in this scenario few doctors treat highly selected, fit patients for a limited number of ICU diagnoses such as sepsis or neutropenic colitis. A third approach would be a core training in one specialty and an supplementary education in the other. Best such training should be implemented in major medical centers as these often have a culture of intensive training and sufficient resources to develop and implement novel structures. If successful this process can spread to other tertiary and even secondary medical centers. "Critical Cancer Care" can only be developed as a joint effort by intensivists and oncologists. Depending on the national system it could be a two year secondary subspecialty training after internal medicine/hematology/oncology or internal medicine/intensive care or anesthesiology/intensive care. Training should be organized by both specialities and should consist of theoretical instruction in the new subject and a common practical training including rotations of e.g. six months to non critical cancer care e.g. oncology clinic for intensivists and anesthesiology for the oncologists. This is in contrast to other subspecialties such as neurosurgical or cardiac critical care that are based on skills taught in the "mother" specialty and that are linked to acute medicine and critical care on a daily basis. Also knowledge relevant to the treatment of cardiologic patients is more familiar to intensivists than the treatment of solid tumors.

To measure the impact of such a structure pilot medical centers should determine the ICU length of stay, respirator days and in hospital mortality as well as patient and referring oncologist satisfaction. The period prior to the implementation of this specialty program should be compared to the period thereafter.

Whichever approach will demonstrate successful it is clear that the specialty of *'critical care of cancer patients' *needs to be recognized, defined and developed. The foreseeable development of cancer medicine necessitates that qualified critical care will eventually be available for a rapidly growing number of cancer patients.

4. Most patients with advanced cancers are treated in an outpatient setting by medical oncologists. However, inpatient treatment is often performed by *physicians specialized in defined organ systems *such as urologists, pulmologists, gastroenterologists, gynecologists etc. It appears to be most beneficial for the critically ill cancer patients to bring these specialties into the above scenario e.g. by regular consultations. First, they are needed to participate in the treatment as referring oncologists and second they can contribute expertise concerning organ-specific complications such as airway stenosis, bleeding from gastrointestinal tumors, urinary tract obstruction etc.

Taken together, the number of cancer patients needing critical care is dramatically rising and as a consequence the array of open questions regarding the optimal management of such patients is growing. These uncertainties can only be addressed successfully when the oncology and intensive care communities start developing 'critical cancer care' concepts as a long-term, joint effort.

## Summary

Cancer medicine is rapidly improving and cancer is eventually becoming a chronic disease. Oncology is therefore characterized by a growing number of older and medically unfit patients that receive numerous novel drug classes with unexpected side effects. These changes will most likely generate more medically challenging patients in acute distress that need to be considered for intensive care. An intense exchange between intensivists, oncologists, psychologists and palliative care specialists is warranted to communicate the developments in each field in order to improve triage and patient treatment. We postulate that "critical care of cancer patients" needs to be recognized as a medical subspecialty and that there is an urgent need to develop it systematically.

## Competing interests

The authors declare that they have no competing interests.

This manuscript has not previously been presented nor is it currently under consideration elsewhere.

## Authors' contributions

MK, ASV, MBB and MH all wrote the manuscript. All authors read and approved the final manuscript.

## Pre-publication history

The pre-publication history for this paper can be accessed here:

http://www.biomedcentral.com/1471-2407/10/612/prepub
